# Predicting the Direction of Stock Market Index Movement Using an Optimized Artificial Neural Network Model

**DOI:** 10.1371/journal.pone.0155133

**Published:** 2016-05-19

**Authors:** Mingyue Qiu, Yu Song

**Affiliations:** Department of Systems Management, Fukuoka Institute of Technology, Fukuoka, Japan; Kyushu University, JAPAN

## Abstract

In the business sector, it has always been a difficult task to predict the exact daily price of the stock market index; hence, there is a great deal of research being conducted regarding the prediction of the direction of stock price index movement. Many factors such as political events, general economic conditions, and traders’ expectations may have an influence on the stock market index. There are numerous research studies that use similar indicators to forecast the direction of the stock market index. In this study, we compare two basic types of input variables to predict the direction of the daily stock market index. The main contribution of this study is the ability to predict the direction of the next day’s price of the Japanese stock market index by using an optimized artificial neural network (ANN) model. To improve the prediction accuracy of the trend of the stock market index in the future, we optimize the ANN model using genetic algorithms (GA). We demonstrate and verify the predictability of stock price direction by using the hybrid GA-ANN model and then compare the performance with prior studies. Empirical results show that the Type 2 input variables can generate a higher forecast accuracy and that it is possible to enhance the performance of the optimized ANN model by selecting input variables appropriately.

## Introduction

The direction of the stock market index refers to the movement of the price index or the trend of fluctuation in the stock market index in the future. Predicting the direction is a practical issue that heavily influences a financial trader’s decision to buy or sell an instrument. Accurate forecast of the trends of the stock index can help investors to acquire opportunities for gaining profit in the stock exchange. Hence, precise forecasting of the trends of the stock price index can be extremely advantageous for investors [1]. Leung, Daouk [2] hold the view that trading could be made profitable by an accurate prediction of the direction of movement of the stock index. Their work suggested that financial forecasters and traders should focus on accurately predicting the direction of movement so as to minimize the estimates’ deviations from the actual observed values. Mostafa [3] also believes that accurate predictions of the direction of stock price indices are very important for investors. However, the behavior of stock markets depends on many qualitative factors such as political, economic, and natural factors, among many others. The stock markets are dynamic and exhibit wide variation, and the prediction of the stock market thus becomes a highly challenging task because of the highly non-linear nature and complex dimensionality [4, 5]. Forecasting of the financial index is characterized by data intensity, noise, non-stationarity, unstructured nature, high degree of uncertainty, and hidden relationships [6–8].

Previous studies have applied various models in forecasting the direction of the stock market index movement. Huang, Nakamori [9] forecasted stock market movement using support vector machines (SVM), and concluded that the model was good at predicting the direction. Kara, Boyacioglu [10] applied Artificial Neural Network (ANN) and SVM in predicting the direction of the Istanbul stock exchange. Their study proves that the two different models are both useful prediction tools, and ANN is significantly better than the SVM model. Şenol and Özturan [11] applied seven different prediction system models for predicting the direction of the stock market index in Turkey, concluding that ANN could be one of the most robust techniques for forecasting. The ANN model has been popularly claimed to be a useful technique for stock index prediction because of its ability to capture subtle functional relationships among the empirical data even though the underlying relationships are unknown or hard to describe [12, 13]. Application of ANN has become the most popular machine learning method, and it has been proven that such an approach can outperform most conventional methods [14–20]. In this study, we attempt to apply an ANN model to forecast the direction of the Japanese stock market index.

The most popular neural network training algorithm for financial forecasting is the back propagation (BP) algorithm, which is also a widely applied classical learning algorithm for neural networks [21–24]. The BP network has been widely used in the area of financial time series forecasting because of its broad applicability to many business problems and its preeminent learning ability [25]. However, many papers have reported that the ANN model, trained by the BP algorithm, has some limitations in forecasting, and it can easily converge to the regional (local) minimum because of the tremendous noise and complex dimensionality of the stock market data. In view of these limitations, genetic algorithms (GA) has been proposed to overcome the local convergence issue for nonlinear optimization problems. We attempt to determine the optimal set of weights and biases to enhance the accuracy of the ANN model by using GA.

The main objective of this study is to improve the prediction accuracy of the direction of stock price index movement by using the ANN model. First, we focus on the selection of the input variables for forecasting the future trend of the stock market index. The selection of effective indicators that can be used to forecast the output variable of an ANN model is significant prior to modeling. In this study, we compare two basic types of input variables that have been widely used in previous studies to predict the direction of the daily stock market index. To evaluate the performance of these two sets of input variables, the Japanese stock market index is used as an illustrative example. In addition, we improve the prediction accuracy according to the optimization of the learning algorithm of the ANN model. The BP algorithm is a widely applied classical learning algorithm for neural networks. However, it has significant drawbacks that need to be improved using other training algorithms. In this study, genetic algorithm (GA) is employed to improve the prediction accuracy of the ANN model and overcome the local convergence problem of the BP algorithm. The empirical results suggest that the proposed method improves the accuracy further for predicting stock market direction, in comparison with previous studies.

The remainder of this paper is organized as follows. Section 2 describes the ANN model trained by the BP algorithm, and the improvement using the GA. Then, we plot the data and showcase two basic types of input variables that are used to forecast the direction, and the procedure of predicting the stock market direction in Section 3. Section 4 provides the experimental results and compares it with similar studies. Finally, Section 5 presents the discussion and conclusion. Formulas and summary of the statistics for each feature of input variables are given in S1 Appendix.

## Prediction Model

### Artificial neural network (ANN) model

**Fig 1:**
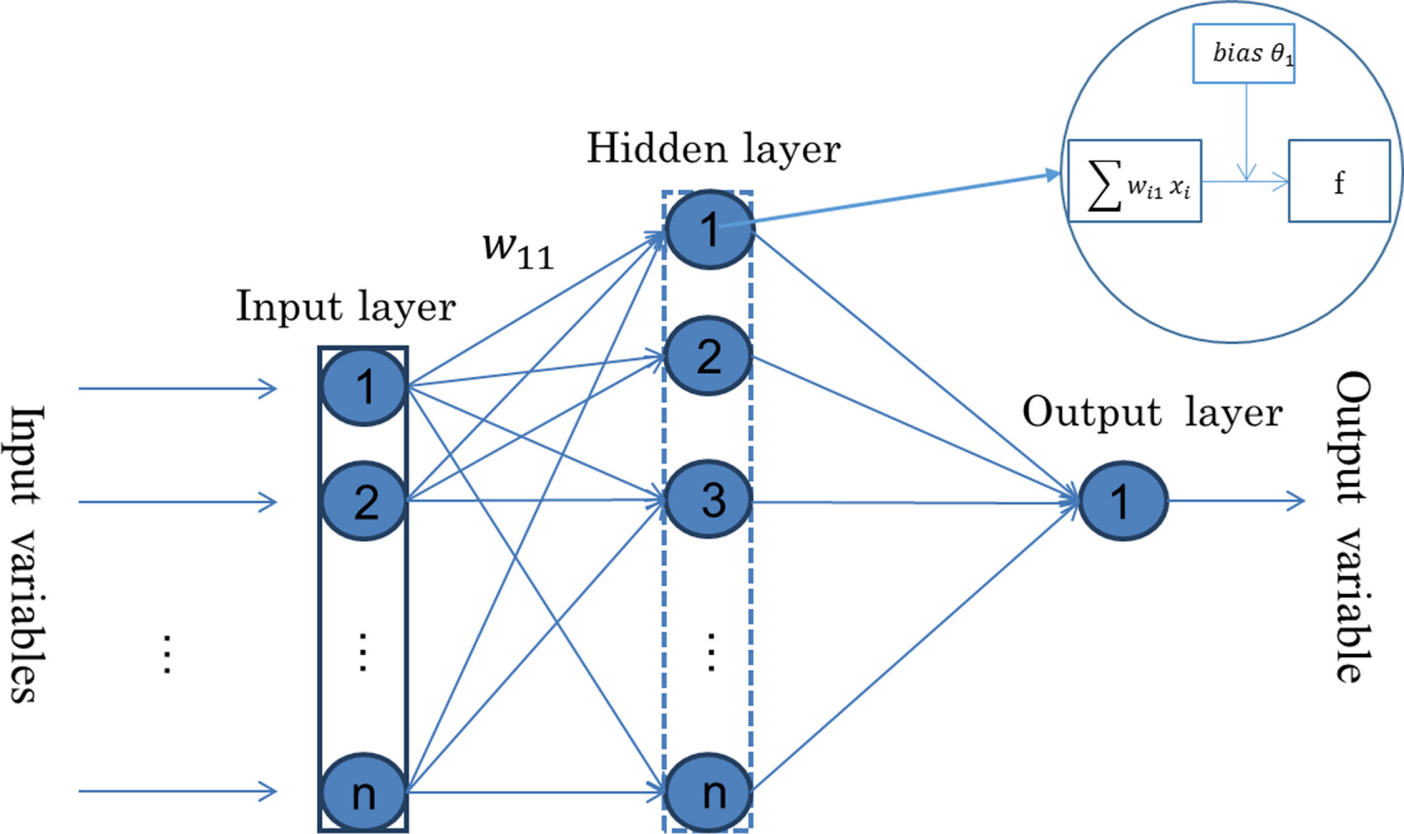
The architecture of the back propagation neural network.

Funahashi [26], Hornik, Stinchcombe [27] have shown that neural networks with sufficient complexity could approximate any unknown function to any degree of desired accuracy with only one hidden layer. Therefore, the ANN model in this study consists of an input layer, a hidden layer, and an output layer, each of which is connected to the other in the same sequence as listed here. The architecture of the ANN is shown in Fig 1. The input layer corresponds to the input variables. We analyze two basic types of input variables for comparing the forecasting accuracy. The hidden layer is used for capturing the nonlinear relationships among variables. In this study, the output layer consists of only one neuron that represents the predicted direction of the daily stock market index.

### Back propagation neural network

The BP algorithm is a widely applied classical learning algorithm for neural networks [22, 23]. As shown in Fig 1, the BP process determines the weights for the connections among the nodes (e.g., *W*_11_ denotes the weight between Node 1 of the input layer and Node 1 of the hidden layer) and their biases (e.g., *θ*_1_ denotes the bias of Node 1 in the hidden layer) on the basis of the training data. The network weights and biases are assigned initial values first, and the error between the predicted and actual output values is back-propagated via the network for updating the weights and biases repeatedly [28]. When the error is less than a specified value or when the termination criterion is satisfied, training is considered to be completed and the weights and bias values of the network are stored. Detailed descriptions of using the BP algorithm for training the ANN model can be found in Ref. [29].

Although researchers have commonly trained the ANN model by using the gradient technique of the BP algorithm, limitations of gradient search techniques are more apparent when ANNs are applied to complex nonlinear optimization problems [30]. The BP algorithm has two significant drawbacks, i.e., slowness in convergence and an inability to escape local optima [31]. In view of these limitations, global search techniques, such as GA, are proposed to overcome the local convergence problem for nonlinear optimization problems. In this study, we propose to apply the GA technique to optimize the weights and biases of the ANN model, and then predict the direction of the daily closing price movement of the stock market index.

### Improvement using genetic algorithms (GA)

There are many studies that have used GA-based hybrid models to overcome the drawbacks of the BP approach [32–34]. The results of these studies support the notion that GA can enhance the accuracy of ANN models and can decrease the time required for experimentation [35]. In this study, the GA algorithm is utilized to optimize the initial weights and bias of the ANN model. Subsequently, the ANN model is trained by the BP algorithm using the determined weights and bias values.

**Fig 2:**
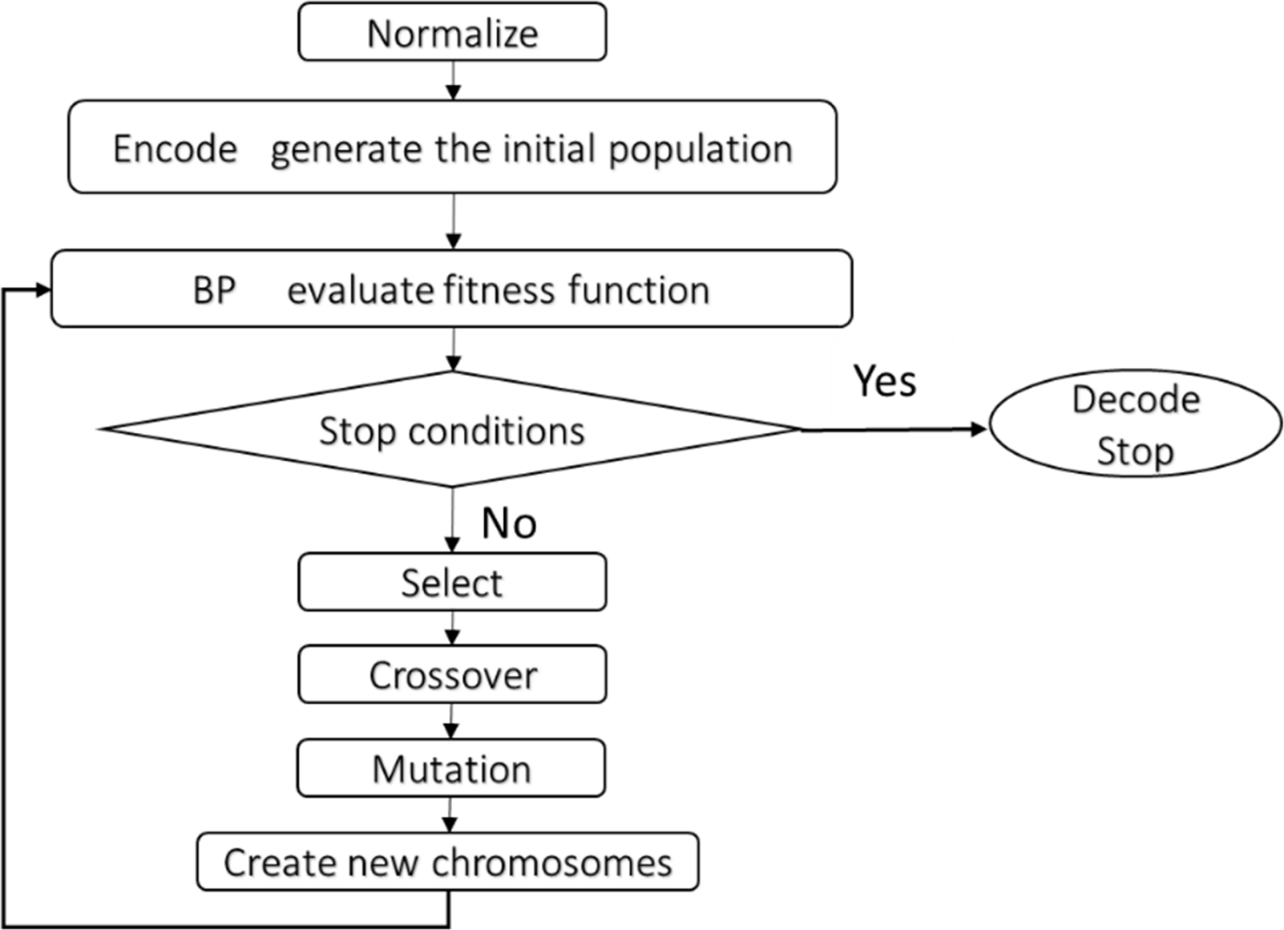
Process flow of the hybrid GA and BP algorithm.

Fig 2 shows the procedural flow of the proposed hybrid GA and BP algorithm.

The algorithm consists of the following steps:

Step 1: Considering the wide range of the data, we normalize it to make sure that the value of all the variables scale down to vary between zero and one. The normalization is carried out as follows:
  $$RN=\frac{R-R_{min}}{R_{max}-R_{min}},$$ (1)
  where *R* is a sample data. *RN* is the normalized value of *R*, *R*_*min*_ is the minimum value of *R* and *R*_*max*_ is the maximum value of *R*.
Step 2: Encode all the weights and biases in a string and generate the initial population. Each solution generated from the GA is called a chromosome (or an individual). The collection of chromosomes is called a population. Here each chromosome describes the ANN with a certain set of weights and bias values.
Step 3: Train the ANN model using the BP algorithm and then evaluate each chromosome of the current population using a fitness function based on the *MSE* (mean squared error) value.
  $$MSE=\frac{1}{N}\sum_{t=1}^{N}{\left(y_{t}-{\hat{y}}_{t}\right)}^{2},$$ (2)
  where *y*_*t*_ denotes the actual value, and ${\hat{y}}_{t}$ is the predicted value. The value of the fitness function is inversely proportional to the error.
Step 4: Rank all the individuals using the fitness proportion method and select the individuals with a higher fitness value to pass on to the next generation directly.
Step 5: Apply genetic algorithms (e.g., crossover, mutation) to the current population and create new chromosomes. Evaluate the fitness value of the new chromosomes and insert these new chromosomes into the population to replace worse individuals of the current population. Following this, we get the new population.
Step 6: Repeat Steps 3–5 until the stop criterion is satisfied.

## Experimental Design

### Data

**Fig 3:**
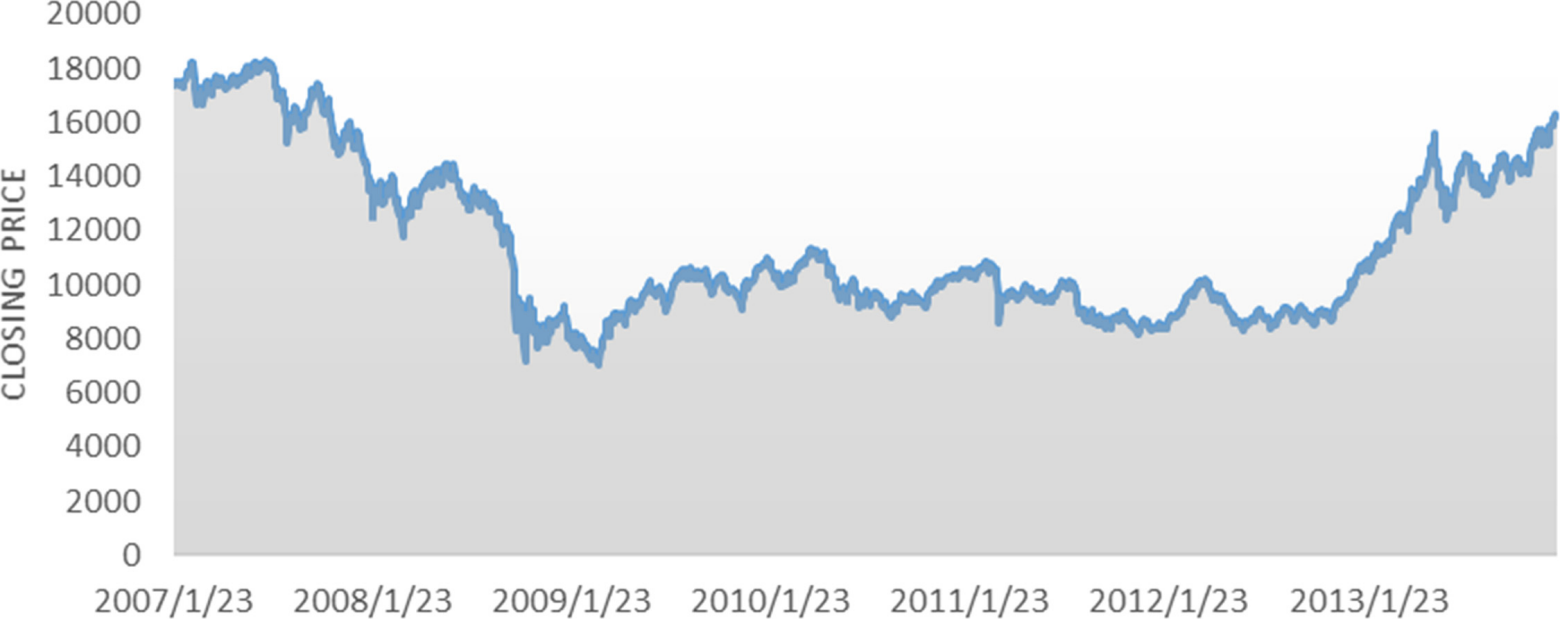
Plots showing the daily Nikkei 225 closing prices from January 23, 2007 to December 30, 2013.

The Nikkei 225 index is the most widely used market index for the Tokyo stock exchange. It includes 225 equally weighted stocks and has been calculated daily ever since 1950. In this study, we attempt to predict the direction of the daily Nikkei 225 index. The research data used in this study are technical indicators that are calculated from the daily price of the Nikkei 225 index. The total number of samples is 1,707 trading days, from January 2007 to December 2013. The total 1,707 data points of the daily Nikkei 225 closing cash prices in the data set are plotted in Fig 3. We divide the entire data into two parts, 78.6% of the data (January 23th, 2007 to October 18th, 2012) is used for in-sample training and 21.4% (October 19th, 2012 to December 30th, 2013) are considered as out-of-sample data. The in-sample data is used to determine the specifications of the model and parameters whereas the out-of-sample data is reserved for the evaluation of the model. The financial data used in this study is obtained from Yahoo Finance.

The original data are normalized before being subjected to the ANN algorithm routine. The goal of linear scaling is to independently normalize each feature component to a specified range. It also ensures that the larger value input attributes do not overwhelm smaller value inputs, which in turn helps decrease prediction errors.

The prediction performance *Hit ratio* is evaluated using the following equation:

$$Hit\;ratio=\frac{1}{n}\sum_{i=1}^{n}P_{i}\;\left(i=1,2,\cdots ,n\right),$$ (3)

where *P*_*i*_ is the prediction result for the *i*^th^ trading day, which is defined by Eq 3. The variable *y*_*t*_ denotes the actual value of the closing stock index for the *i*^th^ trading day, and ${\hat{y}}_{t}$ is the predicted value for the *i*^th^ trading day. The variable *n* denotes the number of test samples.

$$P_{i}=\{\begin{array}{l} 1,\;\left(y_{t+1}-y_{t}\right)\left({\hat{y}}_{t+1}-{\hat{y}}_{t}\right)>0,\\ \;0,\;\text{otherwise}. \end{array}$$ (4)

### Input variables

In the light of previous studies, it is hypothesized that various technical indicators may be used as input variables in the construction of prediction models to forecast the direction of movement of the stock price index [36]. Most financial managers and investors agree on the efficiency of technical indicators and exploit them as a signal for forecasting future trends. On the basis of the reviews of domain experts and prior studies, we notice that most researchers prefer to choose the input variables as shown in Table 1, whereas a few others utilize the variables of Type 2 input variables (shown in Table 2). Tables 1 and 2 list the selected features and their formulas, and we select these technical indicators as the feature subsets based on reviews of prior research studies [11, 37, 38]. One of the aims of this study is to conduct the experiments by using the ANN model with two types of input variables, and then compare the performance of these two experiments with prior studies.

By reviewing previously published studies [7, 10, 11, 25, 39], we set 13 technical indicators as Type 1 feature subset and 9 technical indicators as Type 2 feature subset. Tables 1 and 2 show these indicators, their formulas, and the summary of the statistics for each feature of the two types is presented in S1 Appendix. Technical indicators of the two types of input variables are usually used to predict the future trends, and they are derived from the real stock composite index.

**Table 1:** Selected technical indicators and their formulas (Type 1).

Name of feature	Formulas
Type 1 input variables	
Stochastic %K	(*C*_*t*_ − *L*_*n*_)/(*H*_*n*_ − *L*_*n*_)×100,
Stochastic %D	$\sum_{i=0}^{n-1}\%K_{t-i}/n$,
Stochastic slow %D	$\sum_{i=0}^{n-1}\%D_{t-i}/n$,
Momentum	*C*_*t*_ − *C*_*t*−4_,
ROC (rate of change)	*C*_*t*_/*C*_(*t*−*n*)_ × 100,
LW%R (Larry William’s %R)	(*H*_*n*_ − *C*_*t*_)/(*H*_*n*_ − *L*_*n*_)×100,
A/O Oscillator (accumulation/distribution oscillator)	(*H*_*t*_ − *C*_*t*−1_)/(*H*_*t*_ − *L*_*t*_),
Disparity in 5 days	*C*_*t*_/*MA*_5_ × 100,
Disparity in 10 days	*C*_*t*_/*MA*_10_ × 100,
OSCP (price oscillator)	*MA*_5_ − *MA*_10_/*MA*_5_,
CCI (commodity channel index)	(*M*_*t*_ − *SM*_*t*_)/(0.015 × *D*_*t*_),
RSI (relative strength index)	$100-100/(1+\frac{\sum_{i=0}^{n-1}Up_{t-i}}{\text{n}}/\frac{\sum_{i=0}^{n-1}Dw_{t-i}}{\text{n}})$,

*C*_*t*_ is the closing price and *L*_*t*_ is the lowest price of the Nikkei 225 index at time *t*. *L*_*n*_ is the lowest low price of the Nikkei 225 index in the last *n* days, *H*_*t*_ is the highest price of the Nikkei 225 index at time *t*, *H*_*n*_ is the highest high price of the Nikkei 225 index in the last *n* days. *MA*_*n*_ is the moving average of the price value in the last *n* days: $MA_{\text{n}}=(\sum_{i=1}^{n}C_{t-i+1})/n,\;M_{t}=\frac{H_{t}+L_{t}+C_{t}}{3}$. $SM_{t}=(\sum_{i=1}^{n}M_{t-i+1})/n,\;D_{t}=(\sum_{i=1}^{n}|M_{t-i+1}-SM_{t}|)/n$. *Up*_*t*_ is the upward price change of the Nikkei 225 index at time *t* and *Dw*_*t*_ is the downward price change of the Nikkei 225 index at time *t*.

**Table 2:** Selected technical indicators and their formulas (Type 2).

Name of feature	Formulas
Type 2 input variables	
OBV	*OBV*_*t*_ = *OBV*_*t* − 1_ + Ɵ * *V*_*t*_,
*MA*_5_	$MA_{5}=(\sum_{i=1}^{5}C_{t-i+1})/5$,
BIAS_6_	${\text{BIAS}}_{6}=\left(\frac{C_{t}-MA_{6}}{MA_{6}}\right)\times 100\%$,
*PSY*_12_	*PSY*_12_ = (*A*/12) × 100%,
*ASY*_5_	$ASY_{5}=(\sum_{i=1}^{5}SY_{t-i+1})/5$,
*ASY*_4_	$ASY_{4}=(\sum_{i=1}^{4}SY_{t-i+1})/4$,
*ASY*_3_	$ASY_{3}=(\sum_{i=1}^{3}SY_{t-i+1})/3$,
*ASY*_2_	$ASY_{2}=(\sum_{i=1}^{2}SY_{t-i+1})/2$,
*ASY*_1_	*ASY*_1_ = *SY*_*t* − 1_,

*V*_*t*_ is the volume of trade of the Nikkei 225 index at time t, $Ɵ=\{\begin{array}{c} +1,C_{t}\geq C_{t-1}\\ -1,C_{t}<C_{t-1} \end{array}$. *PSY*_n_ is the ratio of the number of rising periods over the *n* day period. Variable *A* is number of rising days in the last *n* days. *SY*_*t*_ represents the return of the Nikkei 225 index at time *t*, *SY*_*t*_ =(ln *C*_*t*_ − ln *C*_*t*−1_) × 100. *ASY*_*n*_ is the average return in the last *n* days.

### Prediction process

After we finish the work of collecting the real stock composite index data and calculating the two types of input variables that we will compare in the following process, we plug in the data into the optimized ANN model to forecast the future direction of the stock market. We conduct the prediction process as follows: First, we calculate all the indicators for the two types of input variables. Then, we normalize the data to decrease the experimental errors. Before we enter the data into the ANN model, we optimize all the weights and biases of the ANN model using the GA algorithm. After that, we apply two types of indicators for predicting the direction of next day’s movement by the GA-ANN hybrid model. After we finish all the experiments, the performance among the two types of input variable sets is compared with prior reports.

## Experimental Results

### Comparison of the performances between the two types of input variables

For training the GA-ANN hybrid model, we used the in-sample data. In this section, we test the performance of the two sets of input variables by using out-of-sample data, which includes 300 data points. The hybrid model requires a number of parameters that can influence the performance of the model, and these parameters are described here in Table 3.

**Table 3:** Description of parameters that are used in the hybrid model.

Variable	Value	Definition
n	10	number of neurons in the hidden layer of the ANN model
ep	3000	number of iterations for the hybrid model
mc	0.4	momentum constant of the ANN model
l	0.1	value of learning rate of the ANN model
pcro	0.7	crossover rate of the GA-ANN model
pmut	0.2	mutation rate of the GA-ANN model
popu	100	Initial population number of the GA-ANN model

First, we conducted experiments based on the initial parameter setting, which is mentioned in Table 3. Then, we tested the performance of the two types of indicators by changing the different parameter combinations of the GA-ANN hybrid model. Table 4 shows the best performance of each type of input variables. The *hit ratio* denotes the percentage of trials when the predicted direction was correct. From Table 4, we observe that the best hit ratio for forecasting the direction correctly by applying Type 1 input variables is 60.87% and 81.27% for Type 2 input variables. We conclude that Type 2 input variables are more effective in predicting the direction of the daily closing price of the Nikkei 225 index than the Type 1 input variables. We infer that the accurate prediction performance of the ANN model by using Type 2 input variables is useful for investors and can become a good candidate for predicting the direction of next day’s closing price.

**Table 4:** Comparison of the hit ratio between the two types of input variables.

	Type 1	Type 2
*Hit ratio (%)*	60.87	81.27

### Comparison with similar studies

Predicting the direction of the stock market index is an important topic for most investors. There are many studies published in the recent past that focus on the prediction of these movements. Table 5 lists out some of these prior studies that aim to predict the direction of the stock market indices using various methods. The results of this study are also compared with these research studies in Table 5.

**Table 5:** Comparison of our study with prior research reports.

Studies	Methods	Stock market	Hit ratio (%)
Kim and Han [29]	GA feature discretization	Korea	61.70
Leung et al. [2]	Classification model	US, UK, Japan	68 (Nikkei 225)
Huang et al. [9]	SVM	Japan	75
Kara et al. [10]	BPNN	Istanbul	75.74
Our study	GA-ANN hybrid model	Japan	81.27

According to Table 5, we find that the prediction accuracy is significantly different in various studies, and our model is superior to all the other models. Thus, we consider the set of input indicators and the GA algorithm adopted in this study to be more appropriate for prediction.

In light of the previous studies, many researchers have compared ANN with SVM. For example, Kim [21] applied SVM to predict the stock price index, and compare it with the back-propagation neural networks. Their study shows that SVM outperforms BP neural networks in financial time-series forecasting. We suppose that researchers usually focus on the parameter selection of BP neural networks when they compare it with other models. If they combine the selection of input variables and the optimal adjustment of the weights and biases of the ANN model, the optimized ANN model may still provide a promising alternative to stock market prediction.

## Conclusion

In this study, we applied two types of technical indicators to predict the direction of next day’s Nikkei 225 index movement. We adjusted the weights and biases of the ANN model using the GA algorithm and then tested the performance of the GA-ANN hybrid model by applying these two types of input variables and comparing the predictions with actual data. The experiments revealed that Type 2 input variables can provide better performance and the hit ratio for predicting the direction is 81.27%. We also compared the performance of the GA-ANN hybrid model with similar studies and the results showed that our method was more effective and resulted in higher prediction accuracy.

However, the prediction performance of this study may be improved further in three means. The first method is to combine the two types of input indicators, or test a subset of these variables. In addition, we can include a few other variables that may affect the prediction performance. Second, optimal methods other than the GA may also be utilized to adjust the parameters of ANN model. We may even use models based on probabilistic neural networks for predicting the movement of the stock index. Lastly, we could even propose an investment strategy (portfolio) based on the prediction outcomes of this study for future research, practical use and further validation.

## Supporting Information

S1 Appendix (DOCX)
