# Supplementary material for: Predicting the Direction of Stock Market Index Movement Using an Optimized Artificial Neural Network Model
Source: PLoS One. 2016 May 19;11(5):e0155133. doi: 10.1371/journal.pone.0155133 (PMC4873195; doi:10.1371/journal.pone.0155133)
Supplement: S1 Appendix — (DOCX) [file pone.0155133.s001.docx]

## Appendix: Description of the Input Variables

In this appendix, we describe the two types of input variables that are applied to forecast the direction of the Nikkei 225 index in this study.

The descriptive statistics for Type 1 input variables is shown in Table 6. Stochastic oscillator, which includes the *K* line and *D* line, is an indicator that is used for predicting price turning points by comparing the closing price of a security with its price range.

**Table 6. Summary of the statistics (Type 1).**

| Name of feature | Maximum | Minimum | Average | Standard deviation |
| --- | --- | --- | --- | --- |
| Stochastic %K | 100.000 | 0.000 | 53.302 | 33.525 |
| Stochastic %D | 98.731 | 1.450 | 53.287 | 27.594 |
| Stochastic slow %D | 98.229 | 2.121 | 53.269 | 25.485 |
| Momentum | 1492.680 | $-$2196.660 | $-$2.319 | 366.566 |
| ROC | 119.742 | 76.969 | 100.041 | 3.281 |
| LW%R | 100.000 | 0.000 | 46.698 | 33.525 |
| A/O Oscillator | 4.483 | $-$2.919 | 0.577 | 0.936 |
| Disparity 5 days | 113.795 | 86.815 | 99.999 | 1.819 |
| Disparity 10 days | 113.316 | 79.788 | 99.998 | 2.690 |
| OSCP | 0.067 | $-$0.097 | 0.000 | 0.015 |
| CCI | 166.667 | $-$166.667 | 5.408 | 93.381 |
| RSI | 99.875 | 0.000 | 51.831 | 18.234 |

The descriptive statistics for Type 2 input variables are summarized in Table 7. On-balance volume (OBV) measures the buying and selling pressure as a cumulative indicator that adds volume on up days and subtracts volume on down days. It is also an indicator for measuring the positive and negative volume flow. A rising OBV reflects a positive volume pressure that can lead to higher prices. Conversely, falling OBV means that a negative volume pressure can foreshadow lower prices.

$\mathrm{BIAS}_{n}$ measures the divergence of the current stock price from an *n* day simple moving average of the stock prices. Normally, we choose *n* to be 6 days. The value of $\mathrm{BIAS}_{n}$ may be above or below the moving average when the closing price is far away from the average level.

${PSY}_{n}$ is the psychological line, which is a sentiment indicator, and it is designed to consider the influence of the obvious mood of the market and to detect undertones for a trend change.

**Table 7. The summary of statistics (Type 2)**

| Name of feature | | Max | Min | Average | Standard deviation |
| --- | --- | --- | --- | --- | --- |
| OBV | 1769930.000 | | $-$1188269.000 | 255381.867 | 340334.874 |
| ${MA}_{5}$ | 18209.158 | | 7177.696 | 11507.384 | 2947.335 |
| $\mathrm{BIAS}_{6}$ | 18199.282 | | 7211.380 | 11504.454 | 2944.715 |
| ${PSY}_{12}$ | 0.126 | | −0.147 | 0.000 | 0.020 |
| ${ASY}_{5}$ | 0.917 | | 0.000 | 0.514 | 0.132 |
| ${ASY}_{4}$ | 0.048 | | −0.056 | 0.000 | 0.007 |
| ${ASY}_{3}$ | 0.045 | | −0.065 | 0.000 | 0.008 |
| ${ASY}_{2}$ | 0.077 | | −0.068 | 0.000 | 0.010 |
| ${ASY}_{1}$ | 0.085 | | −0.088 | 0.000 | 0.012 |
